# Integrative metabolomics-genomics analysis identifies key networks in a stem cell-based model of schizophrenia

**DOI:** 10.1038/s41380-024-02568-8

**Published:** 2024-04-29

**Authors:** Angeliki Spathopoulou, Gabriella A. Sauerwein, Valentin Marteau, Martina Podlesnic, Theresa Lindlbauer, Tobias Kipura, Madlen Hotze, Elisa Gabassi, Katharina Kruszewski, Marja Koskuvi, János M. Réthelyi, Ágota Apáti, Luciano Conti, Manching Ku, Therese Koal, Udo Müller, Radu A. Talmazan, Ilkka Ojansuu, Olli Vaurio, Markku Lähteenvuo, Šárka Lehtonen, Jerome Mertens, Marcel Kwiatkowski, Katharina Günther, Jari Tiihonen, Jari Koistinaho, Zlatko Trajanoski, Frank Edenhofer

**Affiliations:** 1 Institute of Molecular Biology & CMBI, Department of Genomics, Stem Cell & Regenerative Medicine, University of Innsbruck, Innsbruck, Austria; 2 Institute of Biochemistry and Center for Molecular Biosciences Innsbruck, University of Innsbruck, Innsbruck, Austria; 3 Neuroscience Center, University of Helsinki, Helsinki, Finland; 4 Department of Psychiatry and Psychotherapy, Semmelweis University, Budapest, Hungary; 5 HUN-REN RCNS, Institute of Molecular Life Sciences, Budapest, Hungary; 6 Department of Cellular, Computational and Integrative Biology—CIBIO, University of Trento, Trento, Italy; 7 Department of Pediatrics and Adolescent Medicine, Division of Pediatric Hematology and Oncology, Faculty of Medicine, Medical Center - University of Freiburg, Freiburg, Germany; 8 biocrates life sciences AG, Innsbruck, Austria; 9 Department of Forensic Psychiatry, University of Kuopio, Niuvanniemi Hospital, Kuopio, Finland; 10 A. I. Virtanen Institute for Molecular Sciences, University of Eastern Finland, Kuopio, Finland; 11 Department of Neurosciences, Sanford Consortium for Regenerative Medicine, University of California San Diego, San Diego, USA; 12 Department of Clinical Neuroscience, Karolinska Institutet, and Center for Psychiatry Research, Stockholm City Council, Stockholm, Sweden; 13 Institute of Life Science, University of Helsinki, FI-00014 Helsinki, Finland; 14 Drug Research Program, Division of Pharmacology and Pharmacotherapy, University of Helsinki, Helsinki, Finland; 15 Institute of Bioinformatics, Biocenter, Medical University Innsbruck, Innsbruck, Austria

**Keywords:** Schizophrenia, Stem cells

## Abstract

Schizophrenia (SCZ) is a neuropsychiatric disorder, caused by a combination of genetic and environmental factors. The etiology behind the disorder remains elusive although it is hypothesized to be associated with the aberrant response to neurotransmitters, such as dopamine and glutamate. Therefore, investigating the link between dysregulated metabolites and distorted neurodevelopment holds promise to offer valuable insights into the underlying mechanism of this complex disorder. In this study, we aimed to explore a presumed correlation between the transcriptome and the metabolome in a SCZ model based on patient-derived induced pluripotent stem cells (iPSCs). For this, iPSCs were differentiated towards cortical neurons and samples were collected longitudinally at various developmental stages, reflecting neuroepithelial-like cells, radial glia, young and mature neurons. The samples were analyzed by both RNA-sequencing and targeted metabolomics and the two modalities were used to construct integrative networks in silico. This multi-omics analysis revealed significant perturbations in the polyamine and gamma-aminobutyric acid (GABA) biosynthetic pathways during rosette maturation in SCZ lines. We particularly observed the downregulation of the glutamate decarboxylase encoding genes *GAD1* and *GAD2*, as well as their protein product GAD65/67 and their biochemical product GABA in SCZ samples. Inhibition of ornithine decarboxylase resulted in further decrease of GABA levels suggesting a compensatory activation of the ornithine/putrescine pathway as an alternative route for GABA production. These findings indicate an imbalance of cortical excitatory/inhibitory dynamics occurring during early neurodevelopmental stages in SCZ. Our study supports the hypothesis of disruption of inhibitory circuits to be causative for SCZ and establishes a novel in silico approach that enables for integrative correlation of metabolic and transcriptomic data of psychiatric disease models.

## Introduction

Although schizophrenia (SCZ) is a detrimental neuropsychiatric disorder affecting 0.32–1.0% of the global population [1], very little is known about the pathological mechanisms underlying the disease’s manifestation and progression. The predominant model of SCZ depicts it as a neurodevelopmental disorder, involving fundamental neurobiological alterations to occur prior to the manifestations of symptoms, through the interplay of genetic predispositions and environmental factors [2]. It has been hypothesized that the pathology of the disease is associated with a distorted regulation and response to dopamine and/or glutamate [3, 4]. However, aberrant glutamatergic and dopaminergic neurotransmission alone fails to capture the complexity of the disease’s etiology [5]. Metabolomics has emerged as a promising novel tool for the identification of SCZ-associated metabolites [6–9]. Recent studies revealed altered metabolic profiles in blood serum and plasma from patients with SCZ when compared to control individuals, including different metabolite classes, like amino acids [10–12], phospholipids [13–16], and neuropeptides [17, 18]. Though these observations may lead to the improvement of the disease diagnosis and the discovery of novel biomarkers, the assessment of a putative link between the dysregulated metabolic and transcriptomic profiles and a distorted early human neurodevelopment remains challenging. This is due to both the limited access to patients’ biomaterial and the poor insights into the correlation of transcriptomic and metabolomic data. Induced pluripotent stem cells (iPSCs) developed into a powerful model to investigate early neurodevelopmental aberrations in SCZ patients [19–22]. iPSCs can be derived by reprogramming adult somatic cells from healthy individuals and patients with SCZ [23]. Patient-specific iPSCs carrying the complex genetic makeup of their donors can subsequently be differentiated into appropriate neural models, as a source for transcriptome-wide analysis and metabolic studies [24–26].

Our study aims at exploiting the iPSC technology to establish an integrative network analysis of a presumed correlation between transcriptomics and metabolomics, focusing on early neurodevelopmental changes in SCZ. To this end, we differentiated iPSC lines derived from SCZ patients and control individuals into cortical neurons and examined six developmental stages along the differentiation trajectory. We performed transcriptome-wide analyses and targeted metabolomics and developed an in silico approach to integrate gene expression data with metabolic profiles in a network, offering a more holistic view of cellular dysregulations. Our analysis revealed a distortion of the main γ-aminobutyric acid (GABA) biosynthetic pathway, through the downregulation of glutamate decarboxylation during the rosette maturation stage. Moreover, we found the non-canonical GABA biosynthetic route through putrescine upregulated in the SCZ lines, presumably due to a compensatory mechanism. Our study establishes a novel in silico approach of correlating metabolic and transcriptomic data, unraveling an imbalance in cortical excitatory/inhibitory dynamics manifested during early neurodevelopmental stages in SCZ iPS cell lines.

## Materials and methods

### Cell culture

Eight human iPSC lines were employed in this study (Supplementary Table S1). The cells were cultured using Corning® Matrigel® hESC-Qualified Matrix (Corning, Cat. No. 354277) coated plates with the use of StemMACS™ iPS Brew XF Medium (Miltenyi Biotec, Cat. No. 130-104-368) or Essential 8™ medium (ThermoFisher Scientific, Cat. No. A157001), in antibiotic-free conditions, and maintained at 37 °C, 5% CO_2_. iPSCs were passaged every 3–5 days using either Accutase™ or 0.5 mM phosphate-buffered saline (PBS)/EDTA. Briefly, when passaging the cells with Accutase™, cells were firstly washed with DMEM, 1 ml of Accutase™ was added per 6-well and the cells were incubated at 37 °C for 3–4 min, to ensure proper cell detachment. After the incubation an equal volume of DMEM was added to the well and the cells were collected and centrifuged at 1200 rpm for 3 min at 4 °C. For splitting with PBS/EDTA (ThermoFisher Scientific, Cat. No. 15575020), cells were briefly washed with DMEM, 1 ml PBS/EDTA was added per 6-well and the cells were incubated until they started to roughly dissociate. The EDTA was aspirated and the cells or the cell pellet (when splitting using Accutase), were resuspended in fresh medium supplemented with 10 μM ROCK inhibitor, Y-27632, (Miltenyi Biotec, Cat. No. 130-106-538). The next day the medium was changed back to iPSC medium without ROCK inhibitor. All cell lines were thoroughly characterized for their pluripotency (Supplementary Fig. 1A, B) and were tested frequently for mycoplasma contamination.

### Cortical neuronal differentiation

The generation of cortical progenitors and neurons was performed as described before [27, 28] with minor modifications. Briefly, iPSCs from five 6-wells were collected with Accutase™ and seeded onto an ES-Matrigel coated 12-well. When 100% confluency was reached, StemMACS™ iPS Brew XF Medium was replaced by neural induction medium (NIM; DMEM/F12 Glutamax, Neurobasal, 100 mM L-Glutamine, 0.5 × N-2, 0.5 × B-27 + Vitamin A, 50 μM Non-Essential Amino Acids, 50 μM 2-mercaptoethanol, 2.5 μg/ml insulin, 1 μM dorsomorphine, 10 μM SB431542). The medium was changed every day until the appearance of a tightly packed neuroepithelial sheet (NES). NES was passaged with 0.5 mM EDTA in a ratio of 1:2 or 1:3 to Corning® Matrigel® Growth Factor Reduced (GFR) Basement Membrane Matrix (GFR-Matrigel; Corning, Cat. No. 354230) coated plates. The next day, the medium was switched to neural maintenance medium (NMM; DMEM/F12 Glutamax, Neurobasal, 100mM L-Glutamine, 0.5 × N-2, 0.5 × B-27 + Vitamin A, 50 μM Non-Essential Amino Acids, 50 μM 2-mercaptoethanol, 2.5 μg/ml insulin) and was changed every other day. Upon the appearance of rosettes, 20 ng/ml FGF2 (Peprotech, Cat. No. 100-18C) were added to the medium for four days. On the fourth day of FGF2 treatment, the cells were split again with 0.5 mM EDTA in a ratio of 1:2 to 1:3 onto GFR-Matrigel coated plates. The medium was switched back to NMM and the cortical progenitors were maintained for about 5–10 days until neurons accumulated outside of the rosettes. At this point, cells were passaged with Accutase™, and 50,000 cells/cm² were seeded on poly-L-ornithin/Laminin coated plates for further neuronal differentiation. Alternatively, 2–4 million cells/ml were frozen with neural freezing medium. Neurons differentiated further with half medium changes every two to three days. Samples were harvested at day (d) 0, 7, 16, 27, 50, and 100.

For the DFMO treatment, adherent cell cultures were treated daily with 10 µM DFMO (difluoromethylornithine hydrochloride hydrate; Merck, Cat. No. D193) starting from the first day of differentiation until the collection of cellular pellet and supernatant for mass spectrometry analysis or fixation for subsequent immunocytochemistry (ICC).

### Immunocytochemistry

Cells were fixed in 4% paraformaldehyde (PFA; Sigma, Cat. No. 158127-500G) in PBS solution for 20 min at room temperature (RT). The non-specific binding was blocked with incubation with blocking buffer (3% bovine serum albumin (BSA), 0.2% Triton ×100 in PBS) for 1 h at RT. The primary antibody (Ab) was diluted in the blocking buffer in the recommended concentration and the Ab solution was applied overnight at 4 °C. The following primary Abs were used in the following concentrations: AFP 1:400 (Dako, Cat. No. A000829-2), GAD65/67 1:100 (Abcam, Cat. No. AB183999), GFAP 1:400 (Sigma, Cat. No. G3893-.2 ML), Ki67-VioR667 1:200 (Miltenyi, Cat. No.130-120-422), MAP2 1:1,000 (SynapticSystems, Cat. No.188006), NEUN 1:500 (Sigma, Cat. No. ABN78), OCT3/4 1:200 (Szabo-Scandic, Cat. No. GTX101497-100), PAX6 1:500 (Invitrogen, Cat. No. 42-6600), S100b 1:750 (Abcam, Cat. No. ab52642), SMA 1:500 (Abcam, Cat. No. ab7817), SOX1 1:200 (R&D Systems, Cat. No. AF3369), SOX2 1:500 (R&D Systems, Cat. No. MAB2018), TAU 1:200 (Cell Signaling Technology, Cat. No. 4019), TUBB3 1:1,000 (BioLegend, Cat. No. 801202 and Abcam, Cat.No. ab52623), vGLUT 1:100 (SynapticSystems, Cat. No. 135311). The secondary Ab was diluted 1:500 in 1.5% BSA, 0,2% Triton ×100 in PBS, and the solution was applied for 2 h at RT. The secondary Abs used in this study were: donkey anti-rabbit Alexa Fluor^TM^ 488 (ThermoFisher Scientific, Cat. No. A-21206), donkey anti-rabbit Alexa Fluor^TM^ 546 (ThermoFisher Scientific, Cat. No. A-10040), donkey anti-mouse Alexa Fluor^TM^ 594 (ThermoFisher Scientific, Cat. No. A-21203), donkey anti-mouse Alexa Fluor^TM^ 647 (ThermoFisher Scientific, Cat. No. A-31571), donkey anti-goat Alexa Fluor^TM^ 594 (ThermoFisher Scientific, Cat. No. A-11058), goat anti-chicken Alexa FluorTM 594 (ThermoFisher Scientific, Cat. No. A32759). Finally, the nuclei were counterstained using 4’,6-diamidino-2-phenylindole (DAPI; ThermoFisher Scientific, Cat. No. D21490) in PBS in 1:5000 dilution for 5 min at RT. The coverslips were mounted using Aqua-Poly/Mount mounting medium (PolySciences, Cat. No. 18606-20).

### Microscopy, image acquisition and image analysis

Fluorescent pictures were acquired with the Zeiss Axio Observer Z1 inverted fluorescent microscope and the Leica DMi8 inverted microscope. The image acquisition was performed under the same exposure and laser intensity settings for each set of analyses. For each sample, ten random fields of view were acquired, with a minimum of 20 z-stacks collected per field to ensure proper signal coverage. Further image processing was carried out using the ImageJ software. For quantitative fluorescence intensity analysis, maximum intensity projection was applied and the mean fluorescence intensity values were calculated after background noise subtraction. These values were then normalized to the DAPI+ nuclear area to account for variations in cell density in the different fields of view.

### Reverse transcription quantitative PCR

Total RNA was extracted from cells using TRI Reagent® (Merck, Cat. No. T9424), according to the manufacturer’s instructions. Genomic DNA was removed through treatment with DNase I (Sigma-Aldrich, Cat. No. AMPD1). Subsequently, 1 µg of purified RNA was reverse transcribed into cDNA using the RevertAid RT Reverse Transcription Kit (ThermoFisher Scientific, Cat. No. K1691), following the manufacturer’s guidelines. The expression levels of specific target genes at the mRNA level were quantified via reverse transcription quantitative PCR (RT-qPCR) using the 5× HOT FIREPol EvaGreen qPCR Mix Plus (no ROX) (Solis BioDyne, Cat. No. 08-25-00001-10). Samples were analyzed in technical triplicates to ensure data reliability. Non-template controls (NTCs) were included for each primer pair in every assay to monitor for reagent contamination and primer-dimer formation. To confirm the absence of genomic DNA contamination, random RNA samples were evaluated through gel electrophoresis. The RT-qPCR assays were conducted on the CFX Connect Real-Time PCR Detection System (Bio-Rad). Gene expression levels were normalized to the housekeeping gene ACTB. Relative expression changes were calculated employing the ΔΔCt method [29]. The list of the primers used for RT-qPCR assays is shown in Table 1.

**Table 1:** Primers used for qPCR.

Name	Sequence	Source
*ACTB* Fwd	GCTACGAGCTGCCTGACG	[64]
*ACTB* Rev	GGCTGGAAGAGTGCCTCA	[64]
*GAD1* FWD	GCGGACCCCAATACCACTAAC	Harvard Primer bank
*GAD1* REV	CACAAGGCGACTCTTCTCTTC	Harvard Primer bank
*GAD2* FWD	TTTTGGTCTTTCGGGTCGGAA	Harvard Primer bank
*GAD2* REV	TTCTCGGCGTCTCCGTAGAG	Harvard Primer bank

### Bulk RNA sample collection, quality control, library preparation, and bulk RNA sequencing

Total RNA was isolated from cells at six time points during the cortical differentiation and was prepared for paired-end mRNA sequencing. RNA extraction was performed using the TRI Reagent® (Merck, Cat. No. T9424) according to the manufacturer’s guidelines. Genomic DNA digest was performed with the use of the TURBO DNA-free™ Kit (ThermoFisher Scientific, Cat. No. AM2238). For the library preparation, the Illumina TruSeq RNA Library Prep Kit v2 was used (Illumina, Cat. No. RS-122-2001, RS-122-2002). Quality, as well as concentration of RNA were assessed employing the Agilent RNA 6000 Pico kit (Agilent, cat. no. 5067-1513), Nanodrop, the NEBNext® Library Quant Kit for Illumina® (New England Biolabs, Cat. No. E7630S) and the Qubit RNA Integrity and Quality (IQ) Assay Kit (ThermoFisher Scientific, Cat. No. Q33222). All the kits were used according to the manufacturer’s guidelines. Paired-end sequencing was performed with the NextSeq 500/550 v2 Kit (150 cycles) (Illumina).

### Transcriptomic data pre-processing, heatmap generation, and differential gene expression analysis

Low-quality ends and adapter sequences were trimmed using the wrapper Trim Galore!. Reads were mapped to the human reference genome (GRCh38) using the open-source software STAR [30]. The raw counts were generated with the Hypergeometric Optimization of Motif EnRichment (HOMER) suite [31]. All the subsequent analysis was performed using R [32]. Differential gene expression analysis was performed using the DESeq2 package [33]. Raw counts were normalized using the median of ratios (variance stabilization transformation; vst) [34]. Heatmaps were generated with the ClustVis [35] tool, using the z-score of the vst transcriptomic data for every gene. Gene ontology (GO) enrichment analysis was performed using the ShinyGO 0.76 online tool [36].

A likelihood ratio test (LRT) was used to identify the differentially expressed genes (DEGs) of SCZ and control (CTRL) across the multiple time points of neuronal differentiation [32]. The LRT compared the full model containing the covariates ‘sex’, ‘batch’, ‘time point’, and ‘disease’ with a model reducing the covariates ‘sex’, ‘batch’, and ‘time point’. Statistical values were corrected for FDR using the Benjamini-Hochberg method.

### Weighted gene correlation network analysis (WGCNA) and module-traits relationships

Weighted Gene Correlation Network Analysis (WGCNA) allows the generation of modules that include genes that are co-expressed in the same manner. The vst counts were used to build a co-expression network using the WGCNA [37] package in R [32]. The data were corrected for sex and batch effects using the ComBat function that is implemented in the sva package [38]. The topological overlap measure was calculated using the adjacency matrix. The DynamicTree Cut algorithm, implemented in the WGCNA package, was used to identify the different modules. The gray module contains all the genes that were not assigned to any of the other modules. The module eigengene were calculated. Pearson’s correlation was used to compare modules to each other and to the traits SCZ and the differentiation time points in the adjacency matrix. The top 25% of genes with the highest module membership (MM) were identified as hub genes.

### Gene ontology annotation

Functional enrichment analysis was performed with an input gene ID list using the tool g:GOSt from the g:Profiler [39] R package. Statistical significance was computed and the g:SCS-threshold was corrected at *p* < 0.05.

### Targeted metabolomics, sample collection, and data processing

The cells were washed with 1 ml sterile 1x PBS for 60 s. After the wash, the cells were scraped using 1 ml PBS and the suspension was collected and centrifuged at 4000 rpm for 5 min, at RT. The cell pellets were kept constantly on dry ice and stored at −80 °C until further processing. The cell supernatant was collected after a 24-h incubation, centrifuged at 4000 rpm for 10 min, immediately placed on dry ice and stored at −80 °C. Samples were analyzed using the biocrates MxP® Quant 500 (biocrates life sciences AG, Cat. No. 21094.12). Liquid chromatography-tandem mass spectrometry (LC-MS/MS) was employed to analyze small molecules, including analyte classes such as amino acids, biogenic amines, carboxylic acids, and amino acid-related molecules [40]. Lipid species were measured using flow injection analysis tandem mass spectrometry (FIA–MS/MS). Small molecules were quantified with external 7-point calibrations and internal standards and lipids were quantified by internal standards [41]. The raw data were processed by applying a modified 80% rule to reduce the false positive measurements [42]. The actual missing values, i.e., the values over the level of detection (LOD) for one time point but not for another time point, were uniformly at random imputed with a non-zero value between LOD/2 and LOD. Missing values within one class (i.e., time points and metabolites) were imputed using the arithmetic mean of the class. Batch effects were corrected by centering the data within the groups (i.e., time points) and batches. The performance of the normalization was assessed by plotting the row standard deviations versus the row means and the principal component analysis (PCA). In addition, variancePartition analysis was performed to evaluate the contribution of each individual component of the study design (i.e., time point, batch, and condition), to the measured variation of each metabolite [43].

For metabolite extraction, cell pellets were resuspended in 500 µL ice-cold methanol. Metabolites from supernatants (50 µL) were extracted using 450 µL 8:1 methanol:water. Fully 13C, 15N labeled amino acid standard (Cambridge Isotope Laboratories, Cat. No. MSK-CAA-1) and 6D-gamma hydroxybutyrate (Sigma-Aldrich, Cat. No. 615587) were spiked into samples at the first step of the extraction. After simultaneous proteo-metabolome liquid-liquid extraction [44], protein content was determined from extracted cellular interphases using a Pierce Micro BCA Protein Assay Kit (Thermo Fisher Scientific, Cat. No. 23235). Dried metabolite samples from cell pellets were dissolved in 20 µL 0.1% formic acid (FA) or 50 µL 0.1% FA for the analysis from the supernatant samples. The sample (1 µL) was injected on an Atlantis Premier BEH C18 AX column (1.7 µm, 2.1 × 150 mm, Waters, 186009361) equilibrated at 40 °C using an Acquity Premier UPLC system (Waters). A gradient was run at a flowrate of 0.4 mL/min with mobile phase A (0.1% FA in water) and mobile phase B (0.1% FA in acetonitrile) as follows: 1 min at 1% B, to 40% B in 1 min, 40% B to 99% B in 0.5 min, hold at 99% B for 1.1 min, 99% B to 1% B in 0.1 min followed by 1.8 min of re-equilibration at 1% B. GABA and Glutamate (Glu) were detected using a Xevo-TQ XS Mass spectrometer (Waters) equipped with an electrospray ionization source running in positive mode. The transitions 104–>69 (endogenous GABA), 110–>73 (labeled GABA), 148–>102 (endogenous Glu) and 154–>107 (labeled Glu) were used for quantification. The raw files were processed using MS Quan in waters connect (Waters, V1.7.0.7). The data was further analyzed in R and normalized to the protein content.

### Short time-series expression miner (STEM) analysis of metabolomic and transcriptomic data

To analyze time-related cluster dynamics, the non-parametric clustering algorithm of Short Time-series Expression Miner (STEM) was used [45]. STEM is an online tool that assigns genes or metabolites to significant temporal expression profiles. The Maximum Number of Model Profiles and the Maximum Unit Change in Model Profiles between time points were set to 50 and 2, respectively. Data were normalized to d0. Integrated into the STEM tool is a GO enrichment analysis. All annotations (Biological Process (BP), Molecular Function (MF), and Cellular Component (CC)) were selected and applied. Statistical significance was computed and FDR-corrected at *p* < 0.05.

### Network analysis

The network establishment was based on the gene expression and metabolite level changes across the five successive time point comparisons, along the cortical differentiation. The connectivity information for the initial network was acquired from the publicly available recon3D stoichiometric model data set (available at https://www.vmh.life/#downloadview, retrieved in September 2020) [46]. Ultimately, 51 metabolites and 1135 genes were matched with their corresponding IDs.

Briefly, the construction of the network was performed based on the following steps. Initially, all the reactions associated with any of the target genes were extracted. The metabolites associated with these reactions were identified and the educt-product stoichiometry was applied for every metabolite involved in the network. Subsequently, the reaction data were filtered to extract and proceed only with the genes and metabolites measured in our dataset. The network was further enriched with protein-protein interaction information, derived using the signor database (available at https://signor.uniroma2.it/downloads.php, retrieved in September 2020) [47]. Finally, the network vertices were constructed after examining the unique metabolites and genes, existing in the edge dataset and were further enriched with vertex attributes, such as the vertex type (i.e., gene/metabolite). Log_2_ fold changes (log_2_FC) were converted to a color gradient scale, ranging from blue (indicating a downregulation compared to the previous time point) to red (indicating upregulation).

Extraction of subnetworks from the parental network, was based on assigning membership to the pathways, as defined by the KEGG pathway database, and selecting the subnetwork that included the highest number of differentially expressed genes and metabolites, with the closest degree distribution of the vertices. Pie charts with five equal fractions were used in order to visualize the fold changes occurring across a single metabolite or gene, corresponding to the transitions between two succeeding time points. Additionally, ellipses were used for visualizing the metabolites, while the genes were visualized with circles.

Metabolites that were needed as substantial interconnections between measured metabolites, but were not measured in our dataset, were visualized as small dots. The position for every node was provided as coordinates on a 2D plane. Network visualizations were performed using the R igraph package [48].

## Results

### In vitro cortical differentiation of SCZ and control iPSC lines and sampling

**Fig. 1:**
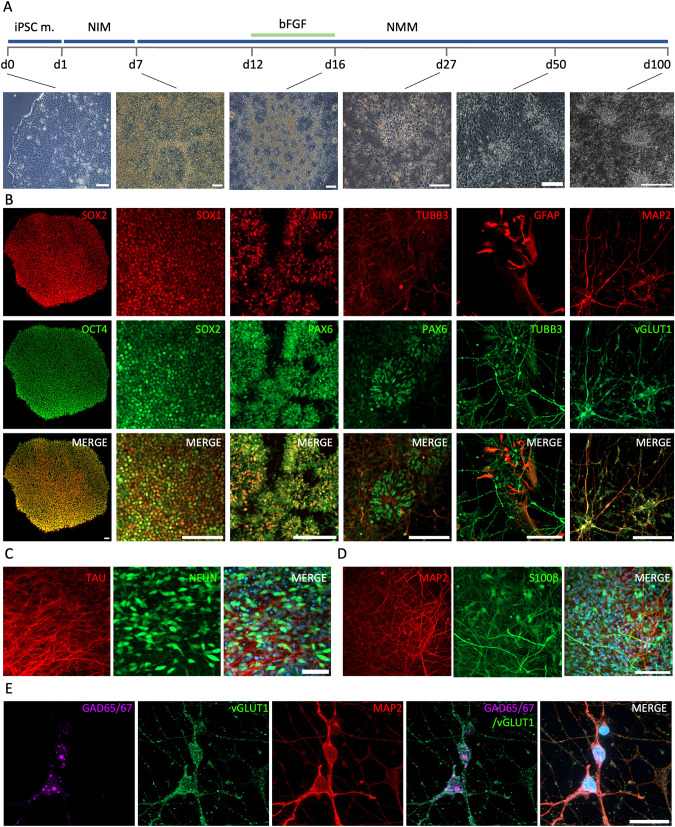
In vitro cortical differentiation of SCZ and CTRL iPSC lines. **A** Schematic presentation of the cortical differentiation protocol (top panel), originally developed by Shi et al. [27] and representative brightfield images (bottom panel) corresponding to the key developmental time points during the cortical differentiation process. Scalebars, 100 μm. **B** Representative immunocytochemistry (ICC) stainings of cell stage-specific markers at day (d)0, 7, 16, 27, 50 and 100 (as depicted in A). Cells at d0 express the pluripotency markers SOX2 (red) and OCT4 (green). Cells on d7 are expressing the neural stem cell markers SOX1 (red) and SOX2 (green). On d16, characteristic rosette structures are formed and the neural progenitors express the proliferation marker KI67 (red) and the neural stem cell marker PAX6 (green). At d27 PAX6+ (green) neural stem cells are still present together with TUBB3+ (red) young neurons. At d50 GFAP+ astrocytes (red) appear together with TUBB3+ (green) neurons. Finally, at d100 mature MAP2+ (red)/ vGLUT1+ (green) neurons are present. Scalebars, 100 µm. **C** ICC analysis showing mature TAU+ (red)/NeuN+ (green) neurons present in culture in the later developmental stages (d100) of neuronal differentiation. Nuclei are counterstained with DAPI. Scalebar, 50 µm. **D** ICC analysis of differentiated cultures at d75 showing S100β+ (green) astrocytes together with MAP2+ (red) mature neurons. Nuclei are counterstained with DAPI. Scalebar, 100 µm. **E** Double-positive GAD65/67 (magenta) and vGLUT1 (green) mature MAP2+ (red) neurons are present in d100 neuronal cultures. Scalebar, 20 µm. iPSC m. induced pluripotent stem cell medium, NIM neural induction medium, NMM neural maturation medium, bFGF basic fibroblast growth factor, d day in vitro.

The reprogramming of adult somatic cells from affected patients into iPSCs allows various approaches for disease modeling [23, 49]. In this study, we subjected iPSCs to a cortical differentiation protocol previously reported by Shi et al. [28] that yields mature cortical neurons in a stepwise manner and particularly reflects very early stages of neurodevelopment (Fig. 1A). We extracted samples from six time points along the neuronal differentiation. At iPSC stage (day 0; d0), cells expressed pluripotency markers, exhibited iPSC characteristic morphology and had the ability to differentiate into the three germinal layers (Fig. 1A, B, Supplementary Fig. 1). Subsequently, the cells were directed to form a tightly packed neuroepithelial sheet (NES)-like structure through the exposure to a neural induction medium (NIM), containing small molecules that modulate the WNT and TGFβ pathway. The neural progenitor cells (NPCs) present at that stage expressed SOX1 and SOX2 (d7) and subsequently traversed through a rosette formation stage (d12). A short treatment with FGF2 yielded in the expansion of the NPC population, expressing the proliferation marker Ki67 and the neural stem cell marker PAX6 (Fig. 1B). Finally, TUBB3+ neurons appeared and started migrating out of the rosettes, observed at around d27, and further differentiated into young and mature neurons (Fig. 1B–D). During the later differentiation time points (d50–100), GFAP+/S100β+ astrocytes appeared in the adherent cultures (Fig. 1B, D). Interestingly, the neurons observed between d50–100 were positive for both the glutamatergic marker vesicular glutamate transporter 1 (vGLUT1), as well as the glutamate decarboxylase 65/67 (GAD65/67) (Fig. 1E), correlating with the recently published finding that certain classes of neurons co-express glutamate and GABA machinery [50].

### Transcriptomic analysis indicates extracellular matrix component abnormalities in SCZ samples

**Fig. 2:**
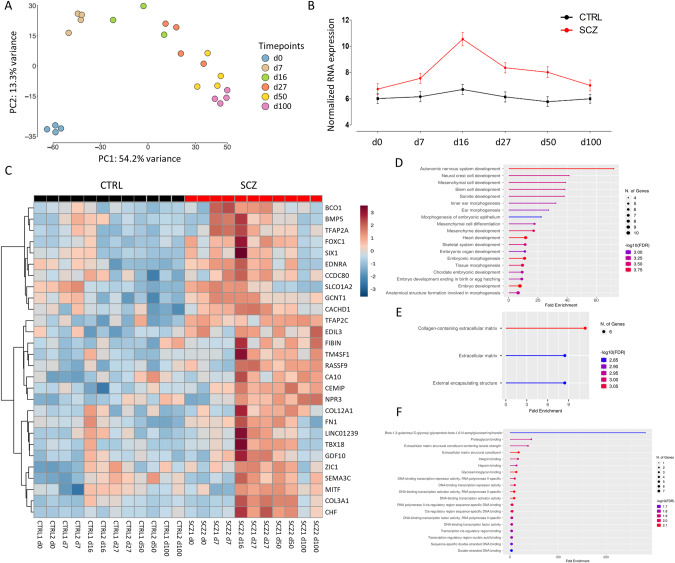
Gene expression analysis of SCZ and CTRL lines during in vitro cortical differentiation. **A** Plot of principal components PC1 and PC2, obtained from the PCA of transcriptomic data from all SCZ and CTRL lines of six time points during cortical differentiation. Each dot represents a cell line, colored by the respective time point. **B** Trajectory analysis of all differentially expressed genes (DEGs) from d0 to 100. Black line, CTRL group; red line, SCZ group. Data are represented as arithmetic mean ± SEM. **C** Heatmap visualization showing the most significant SCZ DEGs. Rows: DEGs; columns: CTRL (CTRL 1 and CTRL 2) and schizophrenia (SCZ 1 and SCZ 2) samples in d0, 7, 16, 27, 50, and 100. GO analysis highlighting the biological processes (**D**), molecular functions (**E**) and cellular components (**F**) of the DEGs. GO terms were generated with the ShinyGO 0.80 graphical gene-set enrichment tool [36]. d day in vitro, CTRL control, SCZ schizophrenia.

In order to investigate the potential dysregulations in SCZ during cortical differentiation at a transcriptional level, we performed bulk RNA sequencing (RNA-seq) of SCZ and CTRL samples derived from distinct time points along the differentiation process (Supplementary Table S2). PCA revealed distinct clustering of all lines at the iPSC stage, followed by a clustering along a developmental trajectory for the subsequent developmental differentiation time points (d0 to d100; Fig. 2A). The largest component of variability (principal component 1, PC1) represents neurogenesis that effectively distinguished the six developmental time points. PC2 (13.3% of explained variance) further separated samples in the iPSC stage from the NPCs and the neural rosettes samples in d7, d16, and d27, and the neuronal cell samples of d50 and d100.

To analyze the genes that are differentially expressed between the CTRL and SCZ lines, DEG analysis was performed revealing 28 genes, all of which were significantly upregulated in SCZ over CTRL (Table 2). Interestingly, trajectory analysis showed the most pronounced DEGs at d16, where 61.2% of the DEGs were upregulated in SCZ as compared to the CTRL group (Fig. 2B). Subsequent analysis of the DEGs revealed that the majority of the upregulated SCZ genes (Fig. 2C) are related to GO terms associated with nervous system development (Fig. 2D) and extracellular matrix (ECM) components (Fig. 2E, F), including genes of the collagen superfamily, as well as *fibronectin 1* (*FN1*), and genes related to DNA-binding-transcription activator activity (Fig. 2C, F). Taken together, transcriptomic analysis indicates upregulated gene transcription associated, among others, with ECM components during SCZ iPSC neural differentiation at all the developmental time points investigated, with a distinct peak at the rosette stage (d16).

**Table 2:** Differentially expressed genes in SCZ.

Gene	Log_2_FC	padj.	Function description
*BCO1*	1.61	3.32E–03	Beta-carotene oxygenase 1
*BMP5*	1.49	3.70E–02	Bone morphogenetic protein 5
*CA10*	1.33	2.53E–02	Carbonic anhydrase 10
*CACHD1*	0.99	2.11E–02	Cache domain containing 1
*CCDC80*	1.39	4.93E–02	Coiled-coil domain containing 80
*CEMIP*	1.29	2.71E–02	Cell migration-inducing hyaluronan-binding protein
*CFH*	1.95	2.40E–03	Complement factor H
*COL12A1*	1.34	4.80E–02	Collagen type XII alpha 1 chain
*COL3A1*	2.27	1.46E–03	Collagen type III alpha 1 chain
*EDIL3*	1.35	3.70E–02	EGF like repeats and discoidin domains 3
*EDNRA*	1.67	1.12E–02	Endothelin receptor type A
*FIBIN*	1.43	4.93E–02	Fin bud initiation factor homolog
*FN1*	1.41	1.84E–02	Fibronectin 1
*FOXC1*	1.88	1.56E–03	Forkhead box C1
*GCNT1*	1.68	1.96E–04	Glucosaminyl (N-acetyl) transferase 1, core 2
*GDF10*	1.52	7.71E–03	Growth differentiation factor 10
*LINC01239*	1.27	2.53E–02	Long intergenic non-protein coding RNA 1239
*MITF*	1.27	1.47E–02	Melanocyte inducing transcription factor
*NPR3*	1.59	2.71E–02	Natriuretic peptide receptor 3
*RASSF9*	1.86	2.05E–04	Ras association domain family member 9
*SEMA3C*	1.27	3.72E–02	Semaphorin 3C
*SIX1*	1.54	1.47E–02	SIX homeobox 1
*SLCO1A2*	1.16	7.71E–03	Solute carrier organic anion transporter family member 1A2
*TBX18*	1.78	3.32E–03	T-box 18
*TFAP2A*	1.58	4.93E–02	Transcription factor AP-2 alpha
*TFAP2C*	1.58	1.56E–03	Transcription factor AP-2 gamma
*TM4SF1*	2.14	1.71E–04	Transmembrane 4 L six family member 1
*ZIC1*	1.24	2.71E–02	Zic family member 1

### WGCNA reveals gene modules correlated to the SCZ trait at rosette stage

**Fig. 3:**
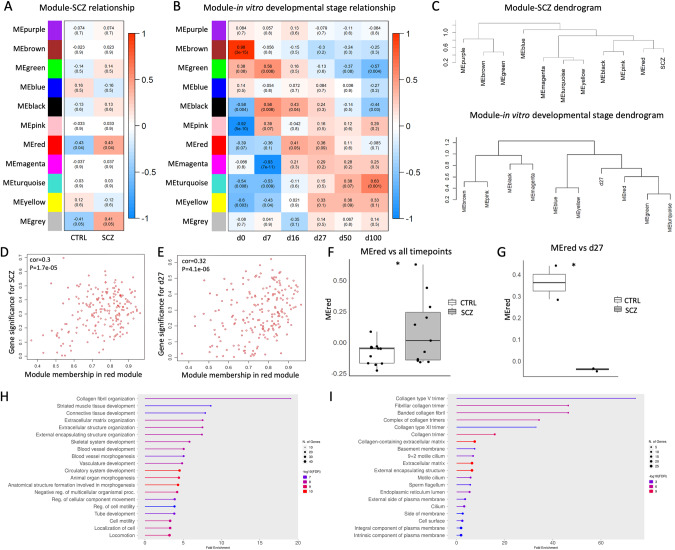
Results of weighted gene correlation network analysis (WGCNA) and module-trait relationships. Module eigengene (ME) comparison of each module with the disease trait (**A**) and the six subsequent time points (**B**), based on Pearson’s correlation. Columns correspond to the different traits; rows correspond to the ME of each module. Upper value in each cell corresponds to Pearson’s correlation; bottom value, *p*-value; right panel, color scale according to correlation. **C** Hierarchical clustering of ME and the SCZ trait reveals higher correlation of the red cluster with SCZ and d27. Scatter plots depicting the gene significance-module membership (MM) correlation for the SCZ trait (**D**) and for d27 (**E**). Box and whiskers plots showing the relationship between the red module and the disease trait (*p*-value = 0.0402; **F**) and the timepoint d27 (*p*-value = 0.0353; **G**). GO analysis highlighting the biological processes (**H**) and cellular components (**I**) of the red module genes. GO terms were generated with the ShinyGO 0.80 graphical gene-set enrichment tool [36]. d day in vitro, CTRL control, SCZ schizophrenia, ME module eigengene. *p < 0.05.

Next, we performed a weighted correlation network analysis (WGCNA) on the bulk transcriptomic data of the two conditions to gain more insights into the biological networks underlying the pathological developmental mechanisms. This analysis identified eleven modules in total, including the gray module (Fig. 3A, B). When the module eigengenes (ME) were compared to the disease trait, the red module was found to be correlated to the SCZ trait (*p-value* = 0.04) (Fig. 3A). Comparison of the six subsequent differentiation time points revealed that the red module is correlated to the rosette stages (d16–27) (Fig. 3B). Dendrogram plots further supported the correlation of the MEred module with the SCZ trait and the d27 timepoint (Fig. 3C). The comparison of the red module membership with the SCZ trait (*p*-value = 1.7e–05; Fig. 3D) and the d27 (*p*-value = 4.1e–06; Fig. 3E) revealed a correlation of 0.3 and 0.32, respectively. Further analysis of the MEred module with all the time points of SCZ vs CTRL supported a significant correlation of the module with the disease trait (*p*-value = 0.0402; Fig. 3F) and the d27 rosette stage (*p*-value = 0.0353; Fig. 3G). Taken together, the WGCNA analysis revealed that the red module hub genes are significantly correlated to the SCZ trait, and to d27, which corresponds to the developmental stage where young neurons are formed and are migrating out of the neural rosettes. Intrigued by the correlation of the red module with the SCZ trait, we sought to determine the GO terms of the red module genes. The red module gene subset was correlated to ECM-associated terms (Fig. 3H), including collagen-associated functions (Fig. 3I). By that, complementary bioinformatics approaches consistently reveal a group of potent gene candidates that are upregulated in the SCZ cell lines and are strongly related with ECM processes, at the late rosette developmental time point (d27).

### Integrative transcriptomic-metabolic in silico analysis allows the generation of system-wide networks

**Fig. 4:**
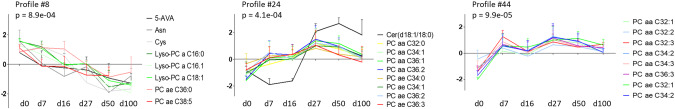
Metabolomic temporal dynamics of SCZ lines during in vitro cortical differentiation. Short time-series expression miner (STEM) plots of significantly enriched temporal profiles obtained from the pre-processed and filtered final metabolomic data set, containing 112 metabolites. All data were normalized against d0. *P*-value, FDR adjusted at *p* < 0.05; y-axis normalized concentration; data are represented as arithmetic mean ± SEM per metabolite from d0–100. d day in vitro, 5-AVA 5-aminovaleric acid, Asn asparagine, Cys cysteine, Lyso-PC lyso-phosphatidylcholine, PC phosphatidylcholine, Cer ceramide.

To assess a presumed SCZ-dependent metabolic aberration, we subjected the samples from the cortical differentiation (Fig. 1A) to a quantitative targeted metabolomics analysis. For that, we harvested both the cell supernatants and the cell pellets and analyzed them employing the MxP® Quant 500 kit (Table S3). This targeted metabolomics approach allows the identification and quantification of 630 metabolites, belonging to 26 analyte classes, including lipids and several small molecules (Table S4). To assess the metabolomic dynamics during the cortical differentiation, a short time-series expression miner (STEM) pattern temporal analysis was performed [45]. STEM analysis revealed three significant profiles, #8, #24, and #44, with *p-value* = 8.9E–4; 4.1E–4, and 9.9E–5, respectively (Fig. 4). These profiles revealed a total of 25 metabolites that were enriched in the SCZ samples. The group of metabolites assigned to profile #8 were two amino acids (AA), asparagine (Asn) and cysteine (Cys), one AA-related metabolite, 5-amino valeric acid (5-AVA), three lyso-phosphatidylcholines (Lyso-PC), Lyso-PC a C16:0, Lyso-PC a C16:1, and Lyso-PC a C18:1, and two phosphatidylcholines (PC), PC ae C36:0, and PC ae C38:5. Profile #8 showed a steadily decreasing trajectory during the neuronal differentiation. Metabolites assigned to profiles #24 and #44 were all PCs with zero to three double bonds in their fatty acid (FA) chain and one ceramide (Cer), Cer (d18:1/18:0). Metabolites assigned to profile #24 increased from d16 to d27 and decreased again at d50. Profile #44 was enriched with metabolite levels increasing from d0 to d7 and stayed relatively stable at later time points (d27 to 100). Taken together, the STEM pattern analysis of metabolites during cortical differentiation revealed a significant enrichment of decreasing trajectories (profile #8) in the CTRL and SCZ groups. Moreover, the analysis revealed 1.64-fold more PCs in the SCZ profiles compared to the CTRL ones.

**Fig. 5:**
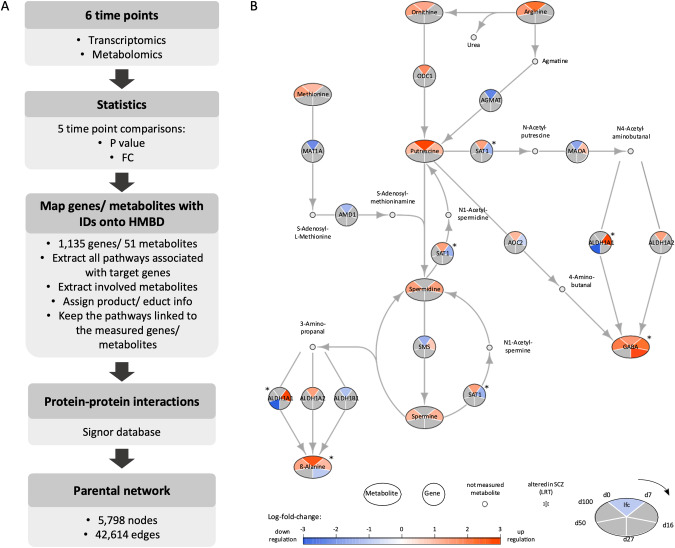
Transcriptomic-metabolomic integrative network reconstruction across neuronal differentiation reveals an interesting subnetwork of polyamine metabolism. **A** Schematic flowchart of the transcriptomic-metabolomic integrative network construction. Transcriptomic and metabolomic data were obtained from the six consecutive time points (see Fig. 1). Five comparisons between every two subsequent time points were performed and the statistical *p*-values and fold change (FC) were calculated. The pathways associated with the measured genes were extracted. The associated metabolites were identified and the product/educt information for each reaction was added. Only the pathways related to the measured genes and metabolites were kept for the network reconstruction. Finally, the parental network was enriched with protein-protein information, extracted from the Signor database and the subnetworks of interest were extracted for further analysis. **B** Polyamine metabolism subnetwork. The global changes in metabolite abundance and gene expression levels, across five consecutive time point comparisons are shown for the subnetwork of the polyamine metabolism. Network nodes depict differentially expressed genes (circles) and metabolites (ellipses), as well as not measured metabolites (small gray dots). Network edges depict individual reactions and the associated genes. Log_2_FC are converted to a color gradient scale, ranging from blue (indicating downregulation to the previous time point) to red (indicating upregulation). The genes and metabolites with no significant change within a certain comparison are depicted in gray. Starting in the upper pie section, the comparison between iPSCs and d7 is depicted, continuing in a clockwise direction for all subsequent comparisons. The genes and metabolites marked with an asterisk are altered in the SCZ condition. FC fold change, lfc log fold change, LRT likelihood ratio test, d day in vitro.

Next, we aimed to obtain an integrative view of the SCZ pathophysiology by transcriptomic and metabolomic analyses and employed Recon3D [46], a metabolic network model for reconstructing networks based on the combination of transcriptomic and metabolomic data. By that, we could investigate the global molecular changes in the different developmental stages of the cortical differentiation, based on the determined alterations in gene expression levels and metabolic abundance. To reconstruct the network, we started with the comparison of DEGs and metabolites between each two subsequent time points of neuronal differentiation yielding five comparisons, as depicted in Table 3 and Fig. 5A. We further compared the identified genes and metabolites to the human metabolome database (HMDB), an electronic database from where we retrieved information about metabolites and genes related to the human metabolism. These results are shown in Table 3 in the row marked as “identified in HMBD”. Next, we reconstructed an initial network where the edges are based on the openly available recon3D stochiometric dataset. 51 metabolites and 1135 genes were retrieved with corresponding IDs. Initially, all the reactions associated with the target genes were extracted. The metabolites associated with the reactions were extracted and the stoichiometry matrix data were applied to add the educt-product information. The reactions that were not associated with genes or metabolites measured in our dataset were removed from the network reconstruction. Finally, the network was enriched with information about protein-protein interactions, obtained from the Signor dataset, resulting in a parental network including 5798 nodes and 42,614 edges (Fig. 5A). Ultimately, here we combined metabolomic with gene expression data generating a parental integrative network that allows the study of global molecular changes that occur during the different in vitro developmental stages of cortical neurogenesis.

**Table 3:** Total number of differentially expressed genes and metabolites between two subsequent time points along the cortical differentiation.

	d0 vs d7	d7 vs d16	d16 vs d27	d27 vs d50	d50 vs d100
Genes					
(*p*_adj_ < 0.1)	5330	2068	571	446	275
Idenitified in HMBD	585	515	82	98	47
Metabolites					
(*p*_adj_ < 0.1)	70	49	33	46	70
Identified in HMBD	24	14	9	6	30

### Integrative transcriptomic-metabolomic network reveals an altered GABA biosynthetic pathway in SCZ

We sought to further examine the combined transcriptional/metabolic pathways, by extracting sub-networks, based on the most converging candidate metabolites and genes in the same pathway, as defined by the HMDB database. Network analysis of the closest connected metabolites and genes pointed towards the polyamine biosynthetic pathway, including dysregulations of putrescine-associated pathways, with distortions of *aldehyde dehydrogenase 1 family member A1* (*ALDH1A1*), as well as the metabolite GABA, in SCZ lines at time points d16–27 (Fig. 5B). GABA is the main inhibitory neurotransmitter in the adult central nervous system (CNS) and its main biosynthetic route occurs through the decarboxylation of glutamate via GAD65/67 [51]. However, the ornithine/putrescine pathway is known to be a non-canonical route for GABA biosynthesis [52]. Along this pathway, ornithine gets decarboxylated to putrescine by ornithine decarboxylase (ODC1; Fig. 5B). Subsequently, putrescine is converted into GABA either by oxidation through AOC2/DAO2, with 4-Aminobutanal as an intermediate, or via an acetyltransferase SAT1- and ALDH1A1-dependent pathway.

**Fig. 6:**
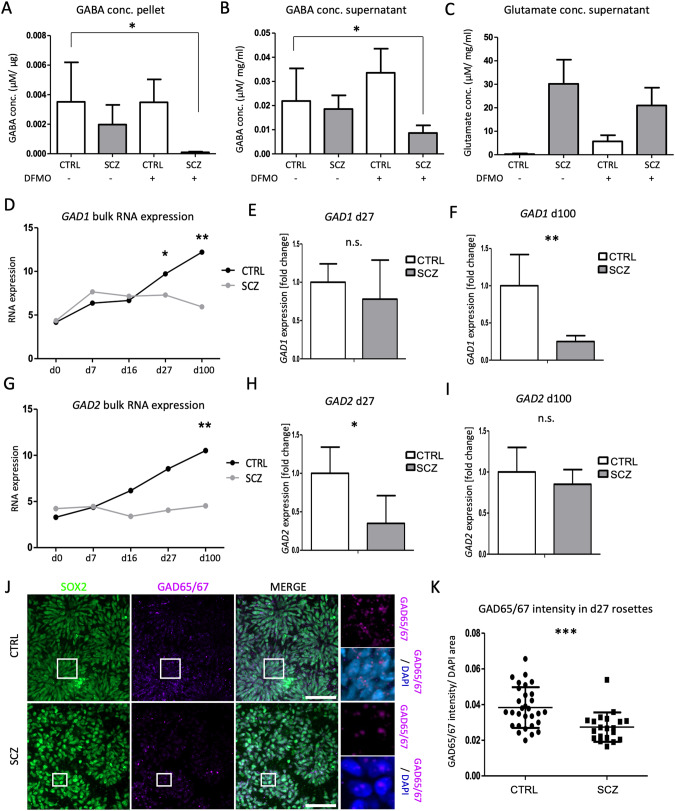
Altered GABA biosynthetic pathways in SCZ samples. Targeted mass spectrometry analysis of GABA levels in cellular pellets (**A**) and supernatants (**B**), as well as glutamate (**C**) from d27 samples, with and without DFMO treatment. Errors bars represent mean ± S.E.M. *GAD1* RNA levels from the bulk RNA-seq data (**D**) and qRT-PCR analyses at d27 (**E**) and d100 (**F**). *GAD2* RNA levels from the bulk RNA-seq data (**G**) and qRT-PCR analyses at d27 (**H**) and d100 (**I**). Errors bars represent mean ± S.D. **J** Representative ICC staining of SCZ and CTRL lines at d27 for the stem cell marker SOX2 (green) and glutamate decarboxylase 65/67 (GAD65/67, magenta). Scalebars, 50 μm. **K** Intensity quantification of GAD65/67. The intensity measurements were normalized against the DAPI+/nuclear area. conc. concentration, n.s. not significant, d day in vitro, CTRL control, SCZ schizophrenia, S.E.M standard error, S.D. standard deviation. *p < 0.05, **p < 0.005, ***p < 0.0001.

Thus, we explored more comprehensively a putative dysregulation of GABA in SCZ samples by targeted LC-MS analyses (Fig. 6). In addition, to investigate the role of putrescine in SCZ GABA biosynthesis we performed a cellular treatment using difluoromethylornithine (DFMO). DFMO is an ODC1 inhibitor that interferes with putrescine biosynthesis [53]. We observed only slightly reduced GABA levels in the SCZ lines compared to the controls. However, DFMO treatment resulted in significantly decreased GABA levels in the SCZ samples in both the cell pellet (Fig. 6A; *p*-value = 0.0363) and the supernatant (Fig. 6B; *p*-value = 0.0494) while the control lines were not affected. This result indicates a stronger reliance of the SCZ lines on the non-canonical putrescine pathway for their GABA biosynthesis. To further investigate this hypothesis, we also analyzed the levels of glutamate, as it is the canonical substrate for GABA production. Indeed, we found glutamate strongly reduced in CTRL lines as compared to SCZ samples, independent from the DFMO treatment (Fig. 6C). Further analysis of the canonical GABA biosynthetic pathway demonstrates that both *GAD1* and *GAD2* mRNA levels are significantly decreased in SCZ lines (Fig. 6D–I), in both d27-mature rosette stage, as well as in d100 neurons. Next, we aimed to further confirm this observation at protein level and performed ICC stainings on d27 samples with both CTRL and SCZ cellular cultures. Indeed, we found GAD65/67 significantly decreased in SCZ samples as compared to the controls (Fig. 6J, K). From these data, we conclude that SCZ cell lines exhibit a distortion of the *GAD1/2*-dependent GABA production in early neurodevelopmental stages, i.e., neural rosette stage, that correlates to neural tube formation in vivo.

## Discussion

Despite collective efforts in the field, pathological mechanisms underlying SCZ pathology remain elusive. The prevalent model depicts SCZ as a neurodevelopmental disorder, involving fundamental neurobiological alterations occurring prior to the manifestations of symptoms, through the interplay of genetic predispositions and environmental factors [2]. At a molecular level, the SCZ pathology is known to be associated with a distorted response to neurotransmitters, including glutamate and dopamine [3]. However, aberrant glutamatergic and dopaminergic neurotransmission alone fails to capture the complexity of the disease’s etiology [4]. Recently, metabolomic studies have proven invaluable in biomarker discovery and in elucidating complex molecular mechanisms. In fact, the metabolome reflects more complex genetic and environmental interactions [54]. For instance, studies in the cancer biology field have successfully employed metabolomic approaches for studying ECM abnormalities [55, 56]. Our integrative transcriptomics-metabolomics study employing SCZ patient-derived iPSCs reveals a GABA distortion in the early rosette maturation stage. GABA is the main inhibitory neurotransmitter, primarily synthesized by decarboxylation of glutamate by GAD56/67 and released by GABAergic interneurons in the adult CNS. Here, we validated our finding by demonstrating a significant reduction of GAD both at RNA and protein levels in SCZ samples. We additionally report a significant reduction of GABA levels in both cellular pellets and supernatants of SCZ samples, indicating deficient GABA biosynthesis in cells derived from SCZ patients. These observations are in line with studies in mice, where *GAD1* neuronal knock-down elicited emotional neuropsychiatric-like abnormalities, as well as in *post-mortem* brain studies from childhood-onset SCZ patients [57, 58]. Moreover, it has been reported that GABA is involved in neural stem/progenitor cell proliferation and differentiation and that it might even exhibit an excitatory function during early development [59–61]. However, GABA dysregulation has not been demonstrated at the early rosette-stage timepoint in human SCZ iPSC-derived cells thus far.

Moreover, our study demonstrates further reduction of GABA levels in DMFO-treated cultures of SCZ iPSC-derived neural cells. Since DMFO inhibits ornithine decarboxylase and by that impacts putrescine biosynthesis, we hypothesize that SCZ cultures partly compensate for the loss of glutamate-based GABA biosynthesis through induction of the non-canonical putrescine pathway. This hypothesis is supported by our integrative network analyses, which underscore SCZ-dependent dysregulations of various enzymes and metabolites of the putrescine/GABA sub-network. We conclude from our data that distorted inhibitory/excitatory imbalances during neurodevelopment of SCZ cells result in partial disruption of the inhibitory circuit formation that is insufficiently compensated at later stages of CNS maturation. In fact, post-mortem studies also indicate an imbalance in the excitatory/inhibitory circuits in SCZ patients [62]. Our findings support the hypothesis that specific defects in the development and function of interneuron progenitors may play a key role in the etiology of psychiatric disorders including SCZ, autism, and intellectual disabilities [62], and assigns GABA a key function in SCZ in this respect.

Finally, we established a new combinatorial transcriptomic-metabolomic network analysis workflow, in order to investigate pathophysiological mechanisms in an integrative, more universal manner. Recently, Wang et al. applied a similar metabolomic-transcriptomic integrative network approach, using data obtained from patient-derived blood samples, in order to identify SCZ biomarkers and to develop a more precise disease diagnosis [63]. In our study, we established a parental integrative network, employing in vitro-derived metabolomic and transcriptomic data. Subsequently, we further elaborated on biologically relevant sub-networks. These approaches can be further used for modeling a vast variety of diseases, including neuropsychiatric disorders.

In conclusion, here we employed an iPSC-based neuronal differentiation model for studying early neurodevelopmental defects in SCZ pathology. Assessment of the metabolome at distinct stages revealed a distortion in the GABA biosynthetic pathways in SCZ lines, a dysregulation observed from the early rosette formation and maturation stages. Therefore, our study elucidates the involvement of GABA dysregulations and compensatory mechanisms during early in vitro neurodevelopment, implying an early imbalance in excitatory/inhibitory circuits. Ultimately, our findings together with the in silico analytical pipeline will contribute to deepen our understanding of SCZ and other psychiatric disorders and potentially build a basis for the development of new therapeutic interventions.

## Supplementary information

Supplementary tables 1-4

Supplementary Figure 1

Supplementary figure legends

## Data Availability

The datasets generated and analyzed during this study are available from the corresponding author upon reasonable request.
