# Supplementary material for: Integrative metabolomics-genomics analysis identifies key networks in a stem cell-based model of schizophrenia
Source: Mol Psychiatry. 2024 Apr 29;29(10):3128–40. doi: 10.1038/s41380-024-02568-8 (PMC11449784; doi:10.1038/s41380-024-02568-8)
Supplement: Supplementary file 1 — Supplementary tables 1-4 [file 41380_2024_2568_MOESM1_ESM.docx]

Supplementary Tables:

**Table S1.** Summary of iPSC lines

| **Cell Line** | **Sex** | **Phenotype** | **Experimental Use** | **Reference** |
| --- | --- | --- | --- | --- |
| Ctrl1 | Male | Control | RNA seq, metabolomics | [1] |
| Ctrl 2 | Female | Control | RNA seq, metabolomics | [2, 3] |
| Ctrl 3 | Male | Control | Metabolomics |  |
| Ctrl 4 | Male | Control | Metabolomics | [4] |
| SCZ 1 | Male | Schizophrenia | RNA seq, metabolomics | [2, 3] |
| SCZ 2 | Female | Schizophrenia | RNA seq, metabolomics | [2, 3] |
| SCZ 3 | Male | Schizophrenia | Metabolomics | [5, 6] |
| SCZ 4 | Male | Schizophrenia | Metabolomics |  |

1. Kwok CK, Ueda Y, Kadari A, Günther K, Ergün S, Heron A, et al. Scalable stirred suspension culture for the generation of billions of human induced pluripotent stem cells using single-use bioreactors. J Tissue Eng Regen Med. 2018;12:e1076–e1087.

2. Tiihonen J, Koskuvi M, Storvik M, Hyötyläinen I, Gao Y, Puttonen KA, et al. Sex-specific transcriptional and proteomic signatures in schizophrenia. Nat Commun. 2019;10.

3. Tiihonen J, Koskuvi M, Lähteenvuo M, Trontti K, Ojansuu I, Vaurio O, et al. Molecular signaling pathways underlying schizophrenia. Schizophr Res. 2021;232:33–41.

4. Mertens J, Paquola ACM, Ku M, Hatch E, Böhnke L, Ladjevardi S, et al. Directly Reprogrammed Human Neurons Retain Aging-Associated Transcriptomic Signatures and Reveal Age-Related Nucleocytoplasmic Defects. Cell Stem Cell. 2015. 2015. https://doi.org/10.1016/j.stem.2015.09.001.

5. Hathy E, Szabó E, Varga N, Erdei Z, Tordai C, Czehlár B, et al. Investigation of de novo mutations in a schizophrenia case-parent trio by induced pluripotent stem cell-based in vitro disease modeling: convergence of schizophrenia- and autism-related cellular phenotypes. Stem Cell Res Ther. 2020;11:1–15.

6. Hathy E, Szabó E, Vincze K, Haltrich I, Kiss E, Varga N, et al. Generation of multiple iPSC clones from a male schizophrenia patient carrying de novo mutations in genes KHSRP, LRRC7, and KIR2DL1, and his parents. Stem Cell Res. 2021;51.

**Table S2.** Sample overview for bulk RNA-seq.

|  | CTRL 1 | CTRL 2 | SCZ 1 | SCZ 2 |
| --- | --- | --- | --- | --- |
| d0 | X | X | X | X |
| d7 | X | X | X | X |
| d16 | X | X |  | X |
| d27 | X | X | X | X |
| d50 | X | X | X | X |
| d100 | X | X | X | X |

**Table S3.** Sample overview for the metabolomic data acquisition.

|  | CTRL 1 | CTRL 2 | CTRL 3 | CTRL 4 | SCZ 1 | SCZ 2 | SCZ 3 | SCZ 4 |
| --- | --- | --- | --- | --- | --- | --- | --- | --- |
| d0 | X | X | X | X | X | X | X | X |
| d7 | X | X | X | X | X | X | X | X |
| d16 | X | X | X | X | X | X | X | X |
| d27 | X | X | X | X | X | X | X |  |
| d50 | X | X | X | X | X | X | X |  |
| d100 | X | X |  |  | X | X |  |  |

**Table S4.** Number of measured analytes per metabolite class and time point after data cleaning using the 80% rule.

| **Metabolite class** | **d0** | **d7** | **d16** | **d27** | **d50** | **d100** |
| --- | --- | --- | --- | --- | --- | --- |
| Amino acids | 17 | 20 | 19 | 20 | 19 | 20 |
| Amino acid related | 2 | 4 | 3 | 3 | 5 | 7 |
| Biogenic amines | 2 | 5 | 5 | 5 | 5 | 5 |
| Carboxylic acids | 1 | 1 | 1 | 1 | 0 | 1 |
| Nucleobases | 0 | 1 | 0 | 1 | 0 | 2 |
| Vitamins | 1 | 1 | 1 | 1 | 1 | 1 |
| Acylcarnitine | 0 | 2 | 2 | 2 | 1 | 3 |
| Fatty acids | 0 | 1 | 0 | 0 | 0 | 2 |
| Lysophosphatidylcholines | 4 | 3 | 3 | 3 | 3 | 7 |
| Phosphatidylcholines_aa | 9 | 16 | 16 | 15 | 22 | 27 |
| Phosphatidylcholines_ae | 3 | 10 | 10 | 7 | 14 | 20 |
| Sphingomyelins | 2 | 2 | 5 | 2 | 5 | 6 |
| Ceramides | 0 | 0 | 1 | 2 | 5 | 7 |
| Hexosylceramides | 0 | 0 | 3 | 0 | 3 | 6 |
| Dihexosylceramides | 0 | 0 | 0 | 0 | 1 | 6 |
| Trihexosylceramides | 0 | 1 | 1 | 0 | 0 | 2 |
| Diglycerides | 0 | 0 | 0 | 0 | 0 | 10 |
| Triglycerides | 0 | 0 | 0 | 0 | 3 | 3 |
| Total | 41 | 67 | 70 | 62 | 84 | 165 |
