# Supplementary figures and images for: Integrative metabolomics-genomics analysis identifies key networks in a stem cell-based model of schizophrenia
Source: Mol Psychiatry. 2024 Apr 29;29(10):3128–40. doi: 10.1038/s41380-024-02568-8 (PMC11449784; doi:10.1038/s41380-024-02568-8)

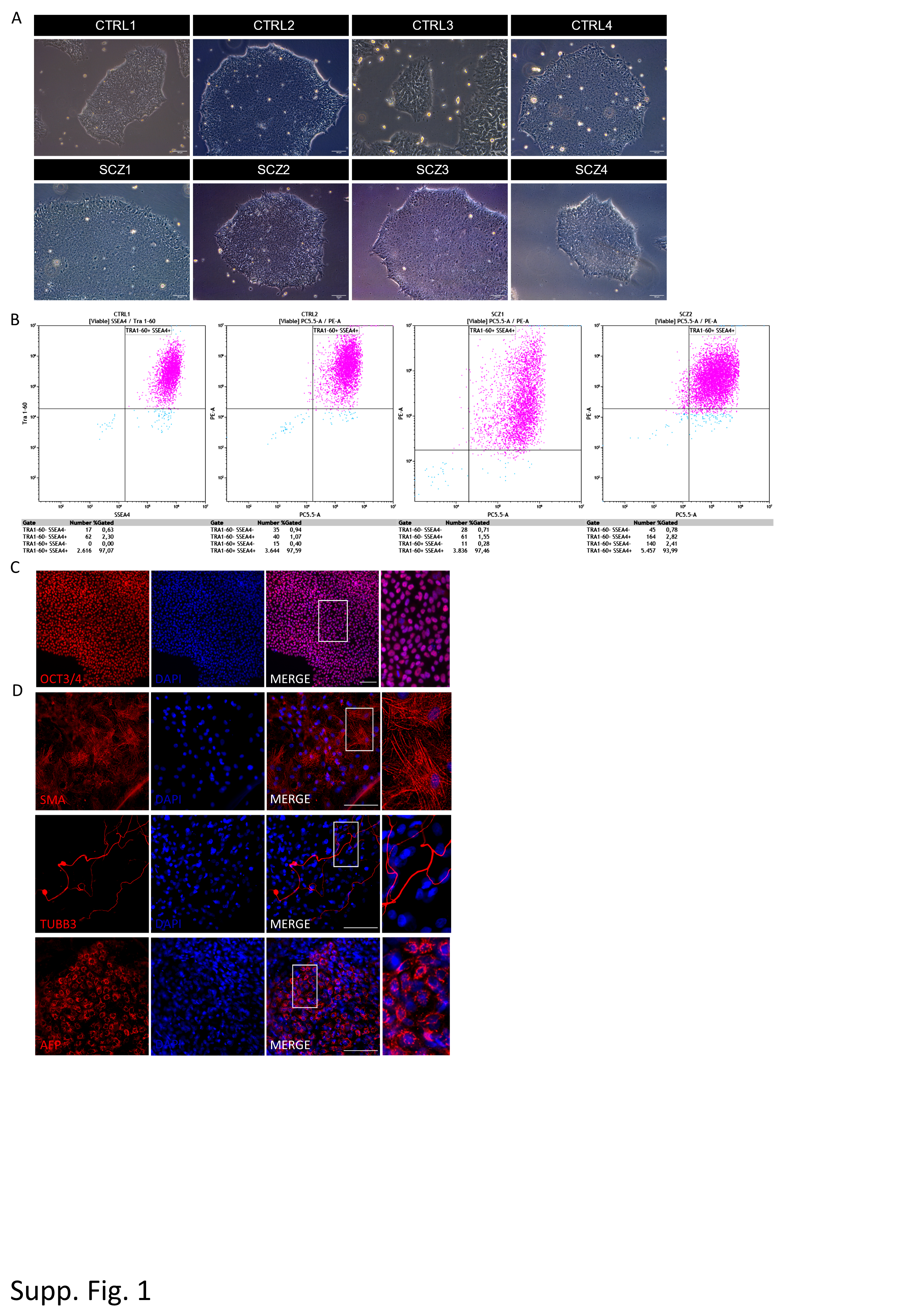

Supplement: Supplementary file 2 — Supplementary Figure 1 [file 41380_2024_2568_MOESM2_ESM.tif]
