# Supplementary material for: Integrative metabolomics-genomics analysis identifies key networks in a stem cell-based model of schizophrenia
Source: Mol Psychiatry. 2024 Apr 29;29(10):3128–40. doi: 10.1038/s41380-024-02568-8 (PMC11449784; doi:10.1038/s41380-024-02568-8)
Supplement: Supplementary file 3 — Supplementary figure legends [file 41380_2024_2568_MOESM3_ESM.docx]

**Supplementary figure legends**

**Supplementary Figure 1.** Validation of pluripotency for the employed iPSC lines. (A) Phase contrast images depicting the iPSC morphology of the used lines. Scalebars, 100 μm. (B) Flow cytometry data depicting the expression of the pluripotency markers SSEA4 and TRA1-60. (C) Representative ICC staining of iPSC lines expressing the pluripotency marker OCT3/4, here shown in red. DAPI was used to counterstain the nuclei. Scalebars, 100 μm. (D) ICC staining after trilineage differentiation of SCZ2 cell line towards the three germ layers. Upper panel: mesoderm, smooth muscle actin (SMA); middle panel: ectoderm, TUBB3; lower panel: endoderm, alpha fetoprotein (AFP). Scalebars, 100 µm.
